# Epigenetics and Neuroinflammation Associated With Neurodevelopmental Disorders: A Microglial Perspective

**DOI:** 10.3389/fcell.2022.852752

**Published:** 2022-05-12

**Authors:** Munekazu Komada, Yuhei Nishimura

**Affiliations:** ^1^ Mammalian Embryology, Department of Life Science, Faculty of Science and Engineering, Kindai University, Osaka, Japan; ^2^ Department of Integrative Pharmacology, Mie University Graduate School of Medicine, Tsu, Japan

**Keywords:** microglia, microRNA, DNA methylation, zebrafish, rodents, fetal alcohol syndrome, autism spectrum disorders, Rett syndrome

## Abstract

Neuroinflammation is a cause of neurodevelopmental disorders such as autism spectrum disorders, fetal alcohol syndrome, and cerebral palsy. Converging lines of evidence from basic and clinical sciences suggest that dysregulation of the epigenetic landscape, including DNA methylation and miRNA expression, is associated with neuroinflammation. Genetic and environmental factors can affect the interaction between epigenetics and neuroinflammation, which may cause neurodevelopmental disorders. In this minireview, we focus on neuroinflammation that might be mediated by epigenetic dysregulation in microglia, and compare studies using mammals and zebrafish.

## Introduction

Neurodevelopmental disorders (NDDs) are characterized by developmental abnormalities in cognition, language, communication, learning, and motor skills (Wilfert et al., 2017; Wamsley and Geschwind, 2020; Savatt and Myers, 2021). NDDs include intellectual disability, learning disorders, autism spectrum disorder (ASD), cerebral palsy, and fetal alcohol syndrome (FAS) (Wozniak et al., 2019; Savatt and Myers, 2021). NDDs are important from both basic and clinical research perspectives (Wilfert et al., 2017; Wamsley and Geschwind, 2020; Díaz-Caneja et al., 2021; Savatt and Myers, 2021). For example, the worldwide prevalence of FAS, ASD, and cerebral palsy is estimated to be 0.75%, 1–2%, and 2%, respectively (Baxter et al., 2015; Stavsky et al., 2017; May et al., 2018). This high prevalence highlights the need to decipher their etiologies and develop early diagnosis and effective treatment strategies. NDDs are considered to be multifactorial (Thapar and Cooper, 2016; Lovely et al., 2017; Wilfert et al., 2017; Díaz-Caneja et al., 2021), but several studies have suggested that convergent pathways exist (Geschwind and State, 2015; de la Torre-Ubieta et al., 2016; Sullivan et al., 2019; Shohat et al., 2021), and dysregulation of neuroinflammation has been reported as a convergent pathway in NDDs (Voineagu et al., 2011; Mottahedin et al., 2017; Cattane et al., 2020; Martino et al., 2020; Panisi et al., 2021).

**FIGURE 1:**
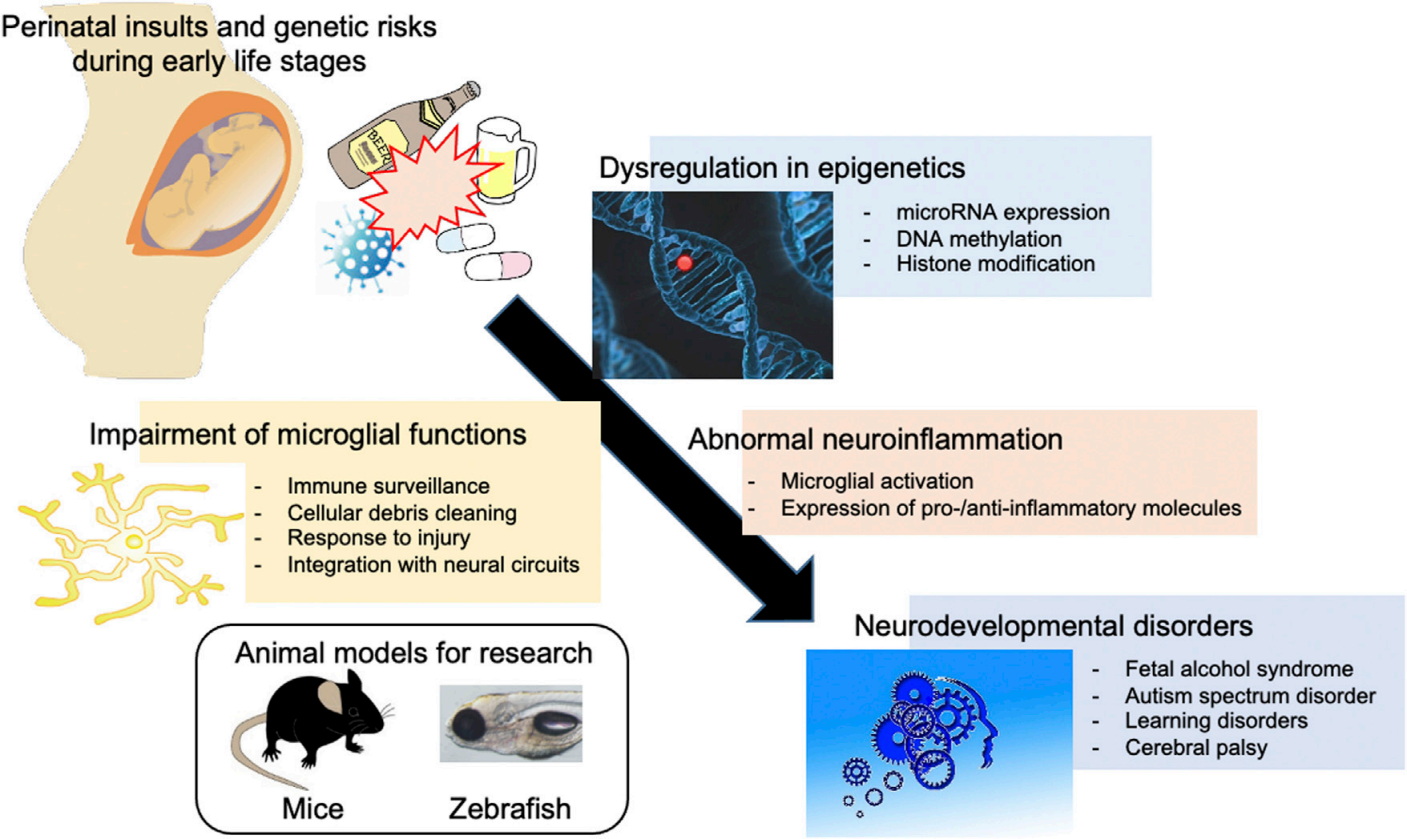
Involvement of environmental and genetic factors in the epigenetic dysregulation and neuroinflammation associated with neurodevelopmental disorders. Perinatal environmental insults such as alcohol and cocaine exposure during development and/or genetic risks such as mutation of *MECP2* and *UHRF1* can cause epigenetic dysregulation in microglia, including the expression of microRNAs important for the development and function of microglia, leading to neuroinflammation and neurodevelopmental disorders. Zebrafish, as well as rodents, can be used to analyze the effect of such factors.

Microglia, resident macrophages of the central nervous system (Ginhoux and Prinz, 2015), play fundamental roles in neuroinflammation (Paolicelli and Ferretti, 2017; Lenz and Nelson, 2018). Microglia can be classified into pro-inflammatory (M1) and anti-inflammatory or alternative activation (M2) phenotypes, although it is now recognized that they exhibit a diverse range of phenotypes (Orihuela et al., 2016; Cheray and Joseph, 2018; Prinz et al., 2019; Stratoulias et al., 2019; Thion and Garel, 2020). The M1 phenotype produces various pro-inflammatory molecules, such as tumor necrosis factor α (TNF), interleukin-1β, -6, and -12, and reactive oxygen species, whereas the M2 phenotype produces anti-inflammatory molecules, such as interleukin-4, -10, and -13 (Orihuela et al., 2016) and neuro-protective and trophic factors, such as insulin-like growth factor 1 and brain-derived neurotrophic factor (Wang et al., 2015). The polarization of microglia into the M1/M2 phenotype is regulated by various epigenetic mechanisms, including DNA methylation, histone modification, and microRNA (miRNA) expression (Kaminska et al., 2016; Cheray and Joseph, 2018). For example, sirtuin 1, a member of the histone deacetylase (HDAC) family, deacetylates various epigenetic regulators, such as E1A binding protein p300, a histone acetyltransferase, and DNA methyltransferase 1 (DNMT1), and promotes M2 polarization (Cheray and Joseph, 2018; van Heesbeen and Smidt, 2019; Wu et al., 2020). In addition, microglia play an important role in brain development through their involvement in neuronal proliferation, survival, neurogenesis, neuronal migration, neural projections, and synaptic plasticity (Li and Barres, 2018). Microglia function is regulated by multiple mechanisms that can be affected by environmental, genetic, and epigenetic factors (Kaminska et al., 2016; Paolicelli and Ferretti, 2017; Cheray and Joseph, 2018). Accumulating evidence suggests that the interaction between epigenetics and neuroinflammation is involved in the etiology of NDDs (Nardone and Elliott, 2016; Boda et al., 2020; Vogel Ciernia et al., 2020).

**TABLE 1:** Reviewed studies on epigenetics and neuroinflammation associated with neurodevelopmental disorders.

Gene	Findings in Mammals	Findings in Zebrafish	References
miR124	PCE causes the promoter hypermethylation and decreases the expression of *miR124* in mouse MG.	The inhibition of *miR124* activates zebrafish MG.	Guo et al. (2016)
	The decrease of *miR124* caused by cocaine exposure post-transcriptionally increases the expression of TLR4 and STAT3 in rat MG.		Periyasamy et al. (2018)
			Chivero et al. (2020)
			Svahn et al. (2016)
miR153	The addition of *miR153* mimic suppress the expression of Tnf in mouse MG.	The expression of *miR153c* is decreased in zebrafish embryo exposed to ethanol	Qiu et al. (2021b)
		Knockdown of *miR153c* causes the phenotype similar to those of zebrafish exposed to ethanol	Tal et al. (2012)
MECP2	Genes associated with the differentially-methylated regions in the brains of Rett syndrome patients show significant enrichment in genes regulated during MG development	The total numbers of mpx-positive neutrophils, but not mpeg-positive MG/macrophages, in the body is increased in the zebrafish model of Rett syndrome	Vogel Ciernia et al. (2020)
	*Mecp2* deficiency causes dysregulation of inflammatory response in mouse microglia		Cronk et al. (2015)
	*Mecp2*-null microglia show the rise of mitochondrial reactive oxygen and the decrease of mitochondrial ATP production in mice		Zhao et al. (2017)
			Jin et al. (2015)
			Van der Vaart et al. (2017)
UHRF1	Knockout of *Uhrf1* causes *Tnf* promoter hypomethylation and increases the expression of Tnf in macrophages, leading to colitis in mice	Knockout of *uhrf1* causes *tnf* promoter hypomethylation and increases the expression of tnf in intestinal epithelial cells, leading to intestinal damage in zebrafish	Qi et al. (2019)
	Intestinal damage activates peripheral immune cells, leading to the breakdown of the blood-brain barrier and dysfunction of MG.		Marjoram et al. (2015)
			Fung et al. (2017)
CSF1R	Knockout of *Csf1r* causes a lack of MG in rat brain	Knockout of *csf1r* cause a lack of MG in zebrafish brains	Patkar et al. (2021)
			Oosterhof et al. (2018)
RNASET2	Knockout of *Rnaset2* causes abnormal activation of MG and increases the expression of interferon-stimulated genes in mouse brain	Knockout of *rnaset2* causes abnormal activation of MG and increases the expression of interferon-stimulated genes in zebrafish brain	Kettwig et al. (2021)
			Hamilton et al. (2020)

MG, microglia; PCE, prenatal cocaine exposure; TLR4, Toll-like receptor 4; STAT3, signal transducer and activator of transcription 3; TNF, tumor necrosis factor α; MECP2, methyl-CpG binding protein 2; ASD, autism spectrum disorder; UHRF1, ubiquitin-like with PHD and ring finger domains 1; CSF1R, colony-stimulating factor 1 receptor; RNASET2, ribonuclease T2.

Rodents have been successfully used to analyze the role of microglia in neuroinflammation associated with NDDs (Johnson and Kaffman, 2018). In mice, primitive microglia derived from yolk sac progenitors (erythromyeloid precursors) migrate into the brain around embryonic day (E) 9.5, where they differentiate into microglia, colonize various brain regions, and regulate neurodevelopment (Prinz et al., 2019; Stratoulias et al., 2019; Thion and Garel, 2020; Sharma et al., 2021). The entry of primitive macrophages and colonization of the brain are also conserved in zebrafish, an alternative animal model for various diseases, including NDDs (Xu et al., 2015; Réu et al., 2017; Ferrero et al., 2018; Bian et al., 2020; Neely and Lyons, 2021). When the second wave of hematopoiesis occurs in mice, microglia progenitors expressing homeobox B8 are generated in the yolk sac, are present in the aorta-gonad-mesonephros (AGM) and fetal liver, and seed into the brain around E12.5 (De et al., 2018). In zebrafish, definitive hematopoiesis begins 15 days post-fertilization in the ventral wall of the dorsal aorta, which is the analogous region of AGM in mammals, leading to the formation of adult microglia in the brain (Xu et al., 2015; Ferrero et al., 2018). Embryonic microglia derived from primitive macrophages gradually disappear in zebrafish (Xu et al., 2015; Ferrero et al., 2018; Sharma et al., 2021). In mice, microglia derived from both primitive and definitive hematopoiesis coexist in the adult brain (Sharma et al., 2021). Despite these differences, the core microglial gene expression signature and microglial functions, such as immune surveillance, cellular debris cleaning, response to injury, and integration with neural circuits, are conserved between mammals and zebrafish (Mazzolini et al., 2020; Neely and Lyons, 2021).

In this minireview, we describe our current understanding of the interaction between epigenetics and neuroinflammation, focusing on microglia in relation to miRNA-124 and 153 (miR124 and miR153), methyl-CpG binding protein 2 (MECP2), and ubiquitin-like with PHD and ring finger domains 1 (UHRF1) (Figure 1). We also compare studies using mammals and zebrafish to provide a future direction for zebrafish-based research on the epigenetic regulation of microglia and neuroinflammation in NDDs (Table 1).

## miR124 and miR153

miRNAs play important roles in the regulation of neurodevelopment by modulating the expression of target genes *via* binding to the 3′-untranslated regions (Thomas et al., 2018). miRNAs are also involved in microglial function (Butovsky and Weiner, 2018; Cheray and Joseph, 2018; Guo et al., 2019; Qiu M. et al., 2021; Zhao et al., 2021). The expression of these miRNAs is epigenetically regulated by prenatal exposure (Knopik et al., 2019; Sushma et al., 2021). Prenatal cocaine exposure (PCE) dysregulates DNA methylation and the expression of miRNAs that are important for the neurodevelopment of offspring (Lambert and Bauer, 2012; Richardson et al., 2015; Vaillancourt et al., 2017). For example, PCE in mice can cause hypermethylation of insulin growth factor II (*Igf2*), leading to decreased expression of *Igf2* in the hippocampus of offspring and impairment of cognitive function (Zhao et al., 2015). PCE also downregulates *miR124* in microglia through promoter hypermethylation in mice (Guo et al., 2016). The cocaine-mediated downregulation of *miR124* in rat primary microglia leads to increased expression of target genes, including Toll-like receptor 4 (TLR4) and signal transducer and activator of transcription 3, and aberrant activation of microglia (Periyasamy et al., 2018; Chivero et al., 2020). Inhibition of *miR124* also activates microglia in zebrafish (Svahn et al., 2016). These studies suggest that the anti-inflammatory role of *miR124* in microglia is conserved between mammals and zebrafish.

Exposure to ethanol during development can have deleterious effects on various cell types, including neurons, oligodendrocytes, astrocytes, and microglia, depending on the dose and timing of exposure and the brain region (Wilhelm and Guizzetti, 2016; Wong et al., 2017; Stratoulias et al., 2019; Almeida et al., 2020; Kane and Drew, 2021; Lussier et al., 2021). The expression of *miR153* is decreased in mouse fetal cerebral cortical-derived neural progenitor cells exposed to ethanol (Balaraman et al., 2012). In microglia located in the hypothalamus of a rat FAS model, the expression is increased (Chastain et al., 2019). TNF secreted from microglia exposed to ethanol can cause neuronal apoptosis and neuroinflammation (Boyadjieva and Sarkar, 2010; Shrivastava et al., 2017). The addition of an *miR153* mimic to mouse microglia suppresses the production of TNF (Qiu T. et al., 2021). In zebrafish exposed to ethanol from 4 to 24 h post-fertilization (hpf), the expression of *miR153c*, a zebrafish homolog of *miR153*, was decreased (Tal et al., 2012). Knockdown of *miR153c* causes phenotypes similar to those of zebrafish exposed to ethanol from 4 to 24 hpf (Tal et al., 2012). Supplementation with folic acid rescued developmental defects in zebrafish FAS models (Muralidharan et al., 2015; Jiang et al., 2020) and ameliorated the dysregulation of miRNA in a mouse FAS model (Wang et al., 2009). Folic acid also affects DNA methylation (Crider et al., 2012). Cocaine exposure decreases the expression of *miR153* in a human neuroblastoma cell line (Cabana-Domínguez et al., 2018). These studies suggest that prenatal substance exposure may affect promoter methylation of miR153 and decrease its expression in both mammalian and zebrafish microglia, leading to neuroinflammation.

## MECP2

Mutation in *MECP2* is the most prevalent cause of Rett syndrome, a progressive NDD with ASD-like features (Amir et al., 1999; Fagiolini et al., 2020). MECP2 is a DNA methylation reader with two major domains: a methyl-binding domain and a transcriptional repressor domain (Fagiolini et al., 2020). MECP2 has a high affinity for methylated CpG (mCG), methylated CpA (mCA), and hydroxymethylated CpA (hmCA), but not for hydroxymethylated CpG (hmCG) (Ip et al., 2018; Connolly and Zhou, 2019; Lavery and Zoghbi, 2019; Tillotson and Bird, 2019). An integrative genome-wide analysis of the methylome and transcriptome using brains from patients with Rett syndrome, idiopathic ASD, and controls revealed that genes associated with the differentially-methylated regions in these NDDs compared with the controls showed significant enrichment in genes regulated during microglial development (Vogel Ciernia et al., 2020). Transcriptome analyses using mouse models have revealed that *Mecp2* deficiency causes dysregulation of the microglial inflammatory response (Cronk et al., 2015; Zhao et al., 2017). *Mecp2*-null microglia also show increased uptake of glutamate, leading to an increase in mitochondrial reactive oxygen species and a decrease in mitochondrial ATP production in mice (Jin et al., 2015). These findings are consistent with other studies demonstrating the dysregulation of neuroinflammation and microglial/macrophage functions in Rett syndrome and ASD (Voineagu et al., 2011; Gupta et al., 2014; O'Driscoll et al., 2015; Parikshak et al., 2016; Schafer et al., 2016; Nance et al., 2017; Kahanovitch et al., 2019; Pecorelli et al., 2020; Marballi and Macdonald, 2021; Wittrahm et al., 2021). Furthermore, these findings suggest that dysregulation of microglia and peripheral immune cells may play pathogenic roles in NDDs and serve as therapeutic targets (Garay and Mcallister, 2010; Reemst et al., 2016; Kaur et al., 2017; Komada et al., 2017; Coomey et al., 2020).

In a zebrafish model of Rett syndrome, a premature stop codon has been introduced before the methyl-binding domain of *mecp2* (Pietri et al., 2013). This zebrafish Rett syndrome model shows increased expression of inflammatory cytokines, impaired locomotion, and decreased anxiety-like behavior, which may be associated with the phenotypes observed in patients with and rodent models of Rett syndrome (Pietri et al., 2013; Van der Vaart et al., 2017). Proteomic analysis using the zebrafish Rett model found that proteins associated with ATP generation and skeletal muscle are dysregulated, which may be associated with impaired motor behaviors in the model (Pietri et al., 2013; Cortelazzo et al., 2017). These findings suggest that MECP2 function is well conserved between zebrafish and mammals. It should be noted, however, that the total number of mpx-positive neutrophils, but not mpeg-positive microglia/macrophages in the body, is increased in the zebrafish model of Rett syndrome (Van der Vaart et al., 2017). Thus, the role of *mecp2* in zebrafish microglia remains unclear.

## UHRF1

The microbiome is involved in the development and maintenance of microglia (Stilling et al., 2014; Erny et al., 2015; Thion et al., 2018; Wang et al., 2018; Erny and Prinz, 2020; Davoli-Ferreira et al., 2021). The densities of microglia in the somatosensory cortex and striatum of germ-free (GF) mice were significantly higher than those of specific-pathogen-free (SPF) mice at E14.5 and E16.5 (Thion et al., 2018). In adults, microglia of GF mice show deficits in the signaling of type I interferon receptors and polarization towards specific phenotypes (Erny et al., 2015). The impairment of microglial maturation is also caused by temporal eradication of the host microbiota or limited microbiota complexity in SPF mice, whereas recolonization with a complex microbiota or supplementation with short-chain fatty acids (SCFA) restores microglial function in GF mice (Erny et al., 2015). SCFA, such as butyrate, propionate, and pyruvate, show inhibitory effects on HDAC activity, suggesting that the function of microglia may be epigenetically regulated by SCFA-producing microbes through the modulation of histone acetylation (Stilling et al., 2014; Fung et al., 2017). Consistent with this idea, genome-wide analysis of chromatin accessibility revealed that there are differentially accessible regions between microglia in GF and SPF mice (Thion et al., 2018). Dysregulation of maternal microbiota caused by maternal infection and exposure to environmental factors during pregnancy can disrupt microglial function and fetal brain development, leading to NDDs (Davoli-Ferreira et al., 2021). The innate immunity regulated by commensal microbiota is conserved in zebrafish (Murdoch and Rawls, 2019).

UHRF1 is a RING E3 ubiquitin ligase that interacts with DNMT1 to copy pre-existing mCG to newly synthesized daughter strands during replication (Li et al., 2021). In mice, knockout of *Uhrf1* decreases mCG at the *Tnf* promoter and increases the expression of *Tnf* in macrophages, which causes colitis, a type of inflammatory bowel disease (IBD) (Qi et al., 2019). In zebrafish, knockout of *uhrf1* decreases mCG at the *tnf* promoter and increases the expression of *tnf* in intestinal epithelial cells, leading to IBD-like intestinal damage (Marjoram et al., 2015). Knockout of *dnmt1* also increases the expression of *tnf* in intestinal epithelial cells (Marjoram et al., 2015). In both models, blocking TNF ameliorates IBD-like phenotypes (Marjoram et al., 2015; Qi et al., 2019). Intestinal damage activates peripheral immune cells, including T_H_17 cells and macrophages, leading to breakdown of the blood-brain barrier and dysfunction of microglia (Fung et al., 2017; Wang et al., 2018; Abdel-Haq et al., 2019; Davoli-Ferreira et al., 2021). These findings suggest that zebrafish is a useful tool for analyzing the gut-microglia connection associated with epigenetics and NDDs.

## Discussion

In addition to the examples discussed above, several studies have demonstrated conserved functions of microglia associated with NDDs in mammals and zebrafish. Leukodystrophies are a group of NDDs characterized by white matter abnormalities (Van der Knaap and Bugiani, 2017). The clinical symptoms include cerebral palsy and cognitive decline (Van der Knaap and Bugiani, 2017). Microglial dysfunction plays an important role in the etiology of leukodystrophy (Garcia et al., 2020; Berdowski et al., 2021). Homozygous mutations in colony-stimulating factor 1 receptor (*CSF1R*) cause pediatric onset leukoencephalopathy (Oosterhof et al., 2019). Homozygous knockout of *CSF1R* homologs in rats and zebrafish causes a lack of microglia in the brain, which is consistent with the findings in humans (Oosterhof et al., 2018; Oosterhof et al., 2019; Patkar et al., 2021). Loss of function mutations in ribonuclease T2 (*RNASET2*) cause early onset leukoencephalopathy resembling congenital cytomegalovirus brain infection in humans (Henneke et al., 2009). Homozygous knockout of *RNASET2* homologs in mice and zebrafish causes abnormal activation of microglia and increased expression of interferon-stimulated genes in the brains (Hamilton et al., 2020; Kettwig et al., 2021; Rutherford et al., 2021). These results warrant further examination to reveal the epigenetic mechanisms underlying leukoencephalopathies using zebrafish models.

Microglia can acquire a specific phenotype depending on the context (Stratoulias et al., 2019), and epigenetics play an important role in the plasticity of microglia (Cheray and Joseph, 2018; Martins-Ferreira et al., 2021). For example, upon stimulation with lipopolysaccharide (LPS), the enhancer of zeste homolog 2, a component of polycomb repressive complex 2 (Prc2), which has histone methyltransferase activity, is increased in mouse microglia, leading to an increase in tri-methylation of histone H3 lysine 27 (H3K27) and pro-inflammatory gene expression through toll-like receptor-induced activation of nuclear factor κB (Nfkb1) (Arifuzzaman et al., 2017; Zhang et al., 2018). The Nfkb1 activation by LPS-TLR4 signaling increases the expression of tet methylcytosine dioxygenase 2 (TET2) and stimulates the expression of LPS-mediated pro-inflammatory cytokines in mouse microglia (Carrillo-Jimenez et al., 2019). TET2 catalyzes the oxidation of 5-methylcytosine (5mC) to 5-hydroxymetylcytosine (5 hmC) (Macarthur and Dawlaty, 2021). 5mC is established *de novo* by two DNA methyltransferases, DNMT3A/B and maintained by DNMT1 (Wu and Zhang, 2014; Lavery and Zoghbi, 2019). Activation of TET2 and/or inhibition of DNMT3A/B decreases mCG levels, resulting in the detachment of MECP2 from genomic mCG sequences (Ip et al., 2018). DNMT3A haploinsufficiency in mice causes behavioral abnormalities and epigenomic dysregulation that overlap with Rett syndrome and ASD (Christian et al., 2020). Notably, the expression of *Mecp2*, *Uhrf1*, *Tet2*, *Dnmt1*, and *Dnmt3a* is also dysregulated in various rodent FAS models (Chen et al., 2013; Kim et al., 2013; Nagre et al., 2015; Varadinova and Boyadjieva, 2015; Veazey et al., 2017; Boschen et al., 2018; Alberry et al., 2021; Lussier et al., 2021). Epigenetic dysregulation caused by exposure to environmental chemicals during development may cause neuroinflammation and NDDs through polarization of microglia into pro-inflammatory phenotypes. Zebrafish are well-suited for analyzing the epigenetic effects of developmental chemical exposure (Aluru, 2017; Cavalieri and Spinelli, 2017).

The phenotypes of microglia are also dependent on the region in which they colonize (Stratoulias et al., 2019; Thion and Garel, 2020). In mice, microglia located in the cerebellum show higher clearance activity than those located in the cerebral cortex or striatum (Ayata et al., 2018). In microglia located in the cerebellar cortex or striatum, PRC2 causes trimethylation of H3K27, resulting in the suppression of gene expression related to clearance activity (Ayata et al., 2018). Regional differences in microglial phenotypes have also been observed in zebrafish (Silva et al., 2021). Epigenetic regulation during development, as well as the ontogeny and function of microglia, is relatively well conserved between zebrafish and mammals (Balasubramanian et al., 2019), making zebrafish a suitable model for analyzing the association between epigenetics, neuroinflammation, and NDDs.
